# APOL1 renal risk variants exacerbate podocyte injury by increasing inflammatory stress

**DOI:** 10.1186/s12882-020-01995-3

**Published:** 2020-08-27

**Authors:** Hidefumi Wakashin, Jurgen Heymann, Hila Roshanravan, Parnaz Daneshpajouhnejad, Avi Rosenberg, Myung Kyun Shin, Maarten Hoek, Jeffrey B. Kopp

**Affiliations:** 1 Kidney Disease Section, NIDDK, NIH, KDB, 10 Center Dr, 3N116, Bethesda, MD 20892-1268 USA; 2 Department of Pathology, The Johns Hopkins University School of Medicine, Baltimore, MD USA; 3 Merck Research Laboratories, Merck and Company, Kenilworth, New Jersey USA; 4 Maze Therapeutics, Redwood City, California USA

**Keywords:** APOL1, Protein isoform, Interleukin-1 beta, NLRP12, Podocytes, Kidney, Chronic kidney disease, Inflammatory stress

## Abstract

**Background:**

Apolipoprotein L1, APOL1, is a trypanosome lytic factor present in human and certain other primates. *APOL1* gene variants, present in individuals of recent sub-Saharan African descent, increase risk for glomerular disease and associate with the disease progression, but the molecular mechanisms have not been defined.

**Objectives:**

We focus on the mechanism how APOL1 variant proteins enhance podocyte injury in the stressed kidney.

**Methods:**

First, we investigated the expression of APOL1 protein isoform and the localization of APOL1 protein in the kidney. Next, we examined the role of APOL1 in the podocyte stress and the inflammatory signaling in the kidney after hemi-nephrectomy.

**Results:**

We identified a novel RNA variant that lacks a secretory pathway signal sequence and we found that the predicted APOL1-B3 protein isoform was expressed in human podocytes in vivo and by BAC-APOL1 transgenic mice. APOL1-B3-G2 transgenic mice, carrying a renal risk variant, manifested podocyte injury and increased pro-IL-1β mRNA in isolated glomeruli and increased IL-1β production in the remnant kidney after uninephrectomy. APOL1-B3 interacted with NLRP12, a key regulator of Toll-like receptor signaling.

**Conclusions:**

These results suggest a possible mechanism for podocyte injury by which one of the APOL1 protein isoforms, APOL1-B3 and its renal risk variants, enhances inflammatory signaling.

## Introduction

African Americans have increased rates of chronic kidney disease, and a major cause of this health disparity are *APOL1* genetic variants, present exclusively in individuals of African descent [1]. APOL1 kill *Trypanosoma brucei*, likely via formation of pores that serve as ion channels [2]. *T. b. rhodesiense* possesses a protein, serum resistance antigen (SRA), which binds *APOL1* and prevents pore formation. The human *APOL1* coding variants, termed G1 and G2, in contrast to the more widespread G0 isoform, elude binding by SRA and kill *T. b. rhodesiense*. The G1 variant has two nonsynonymous coding variants (S342G) and (I384M) and in the G2 variant, N388 and Y389 are deleted.

*APOL1* gene variants are strongly associated with risk for focal segmental glomerulosclerosis (FSGS), HIV-associated nephropathy (HIVAN), and hypertension-attributed kidney disease (arterionephrosclerosis) [1]. *APOL1* risk allele status in deceased donor kidneys affects transplant kidney survival [3], while the *APOL1* risk allele status of the recipient does not affect transplant kidney survival [4]. These data suggest that intra-renal APOL1 expression, rather than circulating APOL1, is associated with kidney disease. As most APOL1 nephropathies are either podocyte diseases (focal segmental glomerulosclerosis, HIV-associated nephropathy) or glomerular and vascular diseases (arterionephrosclerosis), it appears that APOL1 risk variant expression in podocytes and possibly renal vascular cells contributes to kidney disease.

Chronic inflammation contributes to progression of glomerular diseases in an experimental model [5]. Data from the NEPTUNE study of glomerular disease patients indicated a role for APOL1 variants in enhancing glomerular expression of CXCL9 and CXCL11, chemokines induced by proinflammatory cytokines such as TNF and IL-1 [6].

NOD-like receptors (NLR) are pattern recognition receptors that sense intracellular pathogens and host-derived danger signals. Particular NLRs may amplify or suppress the inflammatory response. NLRP3 is a component of the inflammasome, which activates caspase-1 and in turn produces mature IL-1β [7]. Proinflammatory signaling via both NLRP3 inflammasome and NF-κB pathways is involved in the pathogenesis of experimental glomerular disease [5, 8, 9]. NLRP12 is a negative regulator of the proinflammatory signaling pathway induced by Toll-like receptors (TLR) and the tumor necrosis factor (TNF) receptor [10]. NLRP12, which contains pyrin, NACHT, and leucine-rich repeat domains, associates with interleukin-1 receptor associated protein kinase (IRAK1) in canonical NF-κB signaling [10], and associates with TNF receptor-associated factor (TRAF3) and NF-κB inducing kinase in non-canonical NF-κB signaling [11, 12]. NLRP12 negatively regulates the NF-κB and MAPK pathways, as shown in an experimental colitis model [12]. NLRP12 is also a negative regulator of NF-κB signaling in T-cells, and absence of NLRP12 enhances severity of experimental autoimmune encephalomyelitis [13].

APOL1 mRNA comprises seven exons and is expressed in most organs, including the kidney [14–17]. After cleavage of the secretory signal sequence located in exon 4, mature APOL1 is secreted and binds to HDL particles [18]. Isoform C, lacking a signal sequence, is predicted to be retained within the cell. The expression pattern of specific APOL1 splice variants in organs and cells is unknown.

We have examined whether APOL1 regulates proinflammatory signaling in glomerular cells. We identified a novel intracellular protein isoform, APOL1-B3 and found that this isoform associated with NLRP12. Further, APOL1-B3-G2 enhanced podocyte damage in murine nephropathy. This proinflammatory pathway represents a possible therapeutic target for APOL1 nephropathy.

## Methods

### Ethics approvals

Paraffin-embedded autopsy kidney sections were used for immunostaining. Human subjects gave written consent to participate under research protocols approved in advance by the NIDDK Institutional Review Board.

### Cell culture

Conditionally immortalized human podocytes, a generous gift from Moin Saleem, Bristol, UK, were cultured at 33 °C and differentiated at 37 °C with RPMI complete medium with 10% fetal bovine serum, insulin/ transferrin/ selenium, penicillin and streptomycin [19]. HeLa cells were cultured at 37 °C with DMEM complete medium with 10% fetal bovine serum, penicillin and streptomycin.

### Antibodies

APOL1 was detected using the mouse monoclonal antibody (clone CL0171), rabbit polyclonal antibody from Sigma Aldrich (#HPA 018885, St. Louis, MO) and rabbit monoclonal antibody (clone RPR2907) from Abcam, all of which are predicted to recognize isoforms A, B and C, and by rabbit polyclonal antibody, produced at NIDDK, that is specific for isoform B3, affinity purified with immunizing peptides using SulfoLink immobilization kit for peptides (Thermofisher, Carlsbad, CA). Antibody details are provided in the Supplementary Table.

### Quantitative analysis of p57 staining of mouse kidney cortex

Formalin-fixed paraffin-embedded tissue sections were stained with recombinant anti-p57 (Kip2) rabbit monoclonal antibody (Sigma # SAB5500158) which highlights podocyte nuclei with DAB detection on an automated stainer (Lab Vision Autostainer 360, Grand Island, NY). Sections were counterstained with periodic acid Schiff (PAS). Podocyte numbers were assessed as follows: using whole slide scans (Nanozoomer, Hamamatsu, Bridgewater, NJ), (a) 50 glomeruli were randomly selected in each cortical profile and p57 positive podocytes were enumerated, (b) using the PAS stain as the delimiter of the glomerular area, glomerular area was measured using NDPI.view2 (Hammamatsu), and (c) the podocyte number was indexed to the glomerular area (μm^2^). The intensity of podocyte staining was evaluated using the QuPath (qupath.github.io) digital image analysis platform which permits quantification of the stained tissue as follows: (a) At least 40 glomeruli were selected in each cortical region, (b) the software identified the podocytes and graded the diaminobenzidine (DAB) chromogen intensity for each stained pixel on a continuous scale along a pre-defined spectrum considered as “positive stain” by the user, (c) multiple thresholds were tested to explore a range of sensitivities and specificities and the final thresholds were defined based on the highest accuracy to find the region of interest across all the images which permits the comparison of the relative intensities of stains between the images, (d) the average DAB intensity for all pixels inside a podocyte was used to compare the staining intensity of the p57-positive podocytes in the glomerulus across different images.

### Transgenic mice

Human *APOL1* gene locus transgenic mice (BAC/APOL1 mice) were generated using a bacterial artificial chromosome (BAC) which contains the locus for APOL1, resulting in APOL1-G0, −G1 and –G2 mice. A ~ 47 kb human DNA, encompassing only the *APOL1* gene with 5′ and 3′ flanking regions (including exons 1 and 2 of *APOL2* and 3′ region including exons 39–41 of part of *MYH9* gene), was isolated and subcloned from human BAC clone (ENST00000397278, which corresponds to NM_003661). BAC subclone was injected into 129SvJ/B6N F1 embryos and the founders were subsequently backcrossed into 129SvJ. The subclone was sequenced and found to be identical to the reference genome sequences in the NCBI and Ensembl databases, except for the G1 and G2 polymorphisms. For CAG-APOL1-B3 transgenic mice, linear DNA encoding CAG promotor-APOL1-B3 (G0 or G2) FLAG-SV40 polyA signal was introduced into FVB/N mouse embryos by pronuclear injection.

### Mouse nephropathy model experimental procedure

6–8 weeks old, and 2 independent lines of APOL1-B3-G0 transgenic mice and 3 lines of APOL1-B3-G2 transgenic mice were studied, (details in Supplementary tables). Following unilateral nephrectomy, random urine was harvested before and at 3 days after the surgery. Glomeruli were isolated from the remnant kidney at 3 days after the surgery and from intact kidney, by using Dynabeads M-450 Tosylactivated (#14013 Invitrogen) [20]. Animals were euthanized by exposure to compressed carbon dioxide per an NIH-approved animal care and use protocol and per animal facility standard operating procedures.

### Statistics

In vivo data were analyzed by the Kruskal-Wallis test, with post-testing of selected pairs of data sets by Dunnett multiple comparison test (Prism, GraphPad, San Diego, CA). In the figures, group medians are shown. A *p* < 0.05 was accepted as significant.

## Results

### Kidney cells expressed APOL1 splice variants in vitro and in vivo

**Fig. 1:**
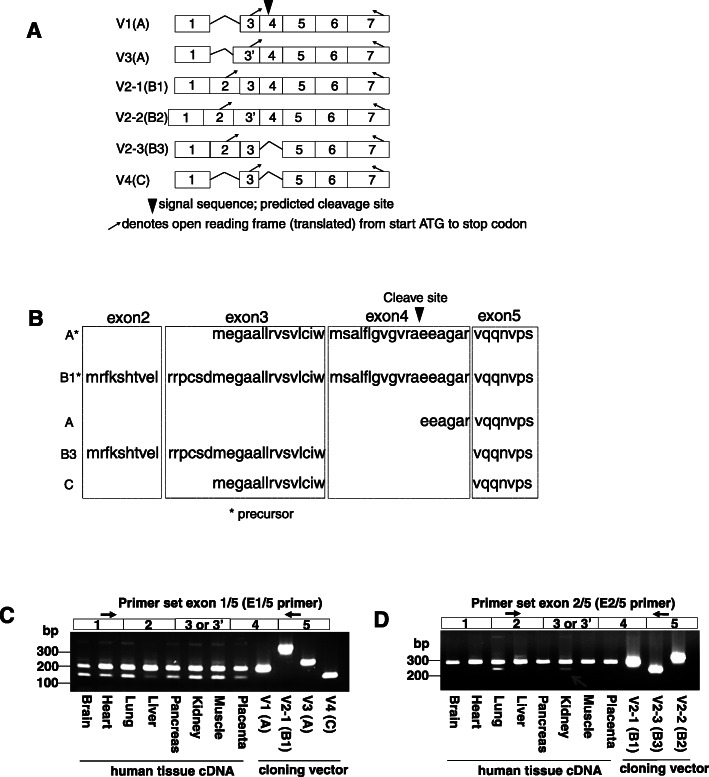
Kidney tissue expressed APOL1 splice variants. **a**, **b** APOL1 splice variants were cloned from differentiated, immortalized human podocytes by TA-cloning. The exons of each of the six mRNA splice variants are shown (**a**), as are the three predicted protein isoforms following signal sequence cleavage (**b**). Exon lengths are not drawn to actual size. **c**, **d** APOL1 splice variant expression in human tissues was analyzed by using a human cDNA library. RT-PCR was performed with two sets of primers as shown by arrows in the above exon schema. APOL1 splice variant TA-vectors were used for PCR positive controls. The major splice variant V1, encoding isoform A, is seen in all examined tissues (**c**). Human lung and kidney expressed the APOL1 splice variant V2–3 (**d**; lower band), encoding protein isoform B3

APOL1 has several mRNA splice variants, predicted to encode diverse protein isoforms. It has not been fully clarified which APOL1 splice variants are expressed in the kidney and which of those contribute to kidney disease. First, we examined the expression of APOL1 mRNA splice variants in immortalized human podocytes by TA-cloning. We identified six mRNA variants, four of them have been described as: V1 (A), V3 (A), V2–1 (B1) and V4 (C); with the protein isoform precursor in parentheses (Fig. 1a)**.** We identified two novel mRNA variants (containing exon 2) which we have named V2–2 (B2) and V2–3 (B3). With regard to V2–2 (B2), we isolated an mRNA encoding exon 2 to exon 7, and partially sharing exon 2 to exon 6 with a variant that has been described in NCBI AceView database (NCBI AceView; APOL1, variant l Aug10). Further, the protein isoforms have predicted variants, as shown by the first number after the letter “V”. Exon 2 is unique to the V2 variants. The APOL1-B3 and APOL1-C protein isoforms are derived from isoform precursors of B3 and C, respectively, and the localization of these proteins are different from isoform A, as they lack a cleavable signal sequence. Thus, APOL1 mRNA splice variants are predicted to produce three different proteins: APOL1-A, APOL1-B3, and APOL1-C (Fig. 1b).

Next, we examined the expression of APOL1 splice variants in human tissues by RT-PCR, using Caucasian cDNA samples with no disease. In kidney, we observed four splice variants, V1, V4 (Fig. 1c), V2–1 and V2–3, while lung also expressed V2–3 (Fig. 1d). We confirmed the presence of APOL1 V2–3 in human kidney by PCR using the V2–3 specific primer pair (Supplementary Figure 1A) and confirmed the DNA sequence of V2–3 PCR product (Supplementary Figure 1C). We then asked whether APOL1 mRNA splicing could also be observed in the mouse kidney. We generated BAC/APOL1 transgenic mice that carry the human APOL1 gene and upstream non-coding regions and we examined the expression of APOL1 splicing variants in the kidneys of the mouse. As shown in Supplementary Figure 1B, APOL1 V1, V2–1 and V2–3 mRNA splice variants were expressed in the kidney, and by using PCR with the V2–1 and V2–3 specific primers, we confirmed the presence of respective DNA sequences (Supplementary Figure 1C).

### APOL1-B3 was expressed in kidney cells but was absent in human serum

Human kidney expressed APOL1 V1, V2–1 and V2–3 mRNAs. APOL1-A, the circulating form, which is mainly expressed in and secreted from liver tissue, is present in human serum and is part of the trypanosome lytic factor. However, localization and function of APOL1-B3 were unknown. To investigate cellular localization of APOL1-B isoforms in kidney tissue, we therefore produced a polyclonal rabbit antibody against a 16 amino acid sequence (MRFKSHTVELRRPCSD) that is encoded by exon 2 and exon 3, is specific to APOL-B isoforms, and is absent from isoforms APOL1-A and APOL1–C. APOL1-B antibodies were affinity purified from the serum of immunized rabbits. We then characterized the specificity of APOL1-B antibody by probing it against the immunizing peptides and APOL1-B protein isoforms. For that, we transfected human podocytes with 3x FLAG-tagged (FLAG-tagged) expression constructs encoding B1, B2 and B3 isoforms. APOL1 protein isoforms were then immunoprecipitated with anti-FLAG antibodies and analyzed by immunoblotting using APOL1-B and mouse anti-APOL1 C-terminal antibodies (clone: CL0171). A signal for APOL1-B was not observed when the APOL1-B antibody was first preincubated with immunizing peptides; this demonstrated that the APOL1-B antibody was specific for the immunizing peptides (Supplementary Figure 2A: top). All three proteins were recognized by the anti-APOL1 C-terminal antibody but only APOL1-B3 was detected by both APOL1-B and APOL1-C antibodies (Supplementary Figure 2A: second from top). This suggests that APOL1-B1 and APOL1-B2 were cleaved, probably in the signal sequence encoded by exon 4 and consequently lost exon 2. APOL1-B3 lacked exon 4 and therefore retained exon 2. Thus, we concluded that the APOL1-B antibody specifically recognized APOL1-B3.

We examined the cellular localization of APOL1-B3 in vivo, using APOL1-B and mouse anti-APOL1 C-terminal antibodies (CL0171). In human serum, APOL1 was not detected by the APOL1-B antibody (Supplementary Figure 2B) but was detected with an APOL1 antibody that is predicted to recognize the C-terminal region of APOL1 A, B and C protein isoforms (Supplementary Figure 2C). APOL1 was originally identified in human serum as two species, polypeptides with apparent molecular weights of 42 kDa and 39 kDa [18]. Amino acid sequence analysis had shown that the 42 kDa APOL1 was the result of cleavage at exon 4, which encodes for the APOL1 signal peptide. The 39 kDa APOL1 species was 26 amino acids shorter at the N terminus than the 42 kDa form and glycosylated [18]. The lower APOL1-B3 protein band was not recognized by APOL1-B antibody (Supplementary Figure 2A), suggesting it was cleaved similar to isoform A. The biological significance of this species is undetermined.

**Fig. 2:**
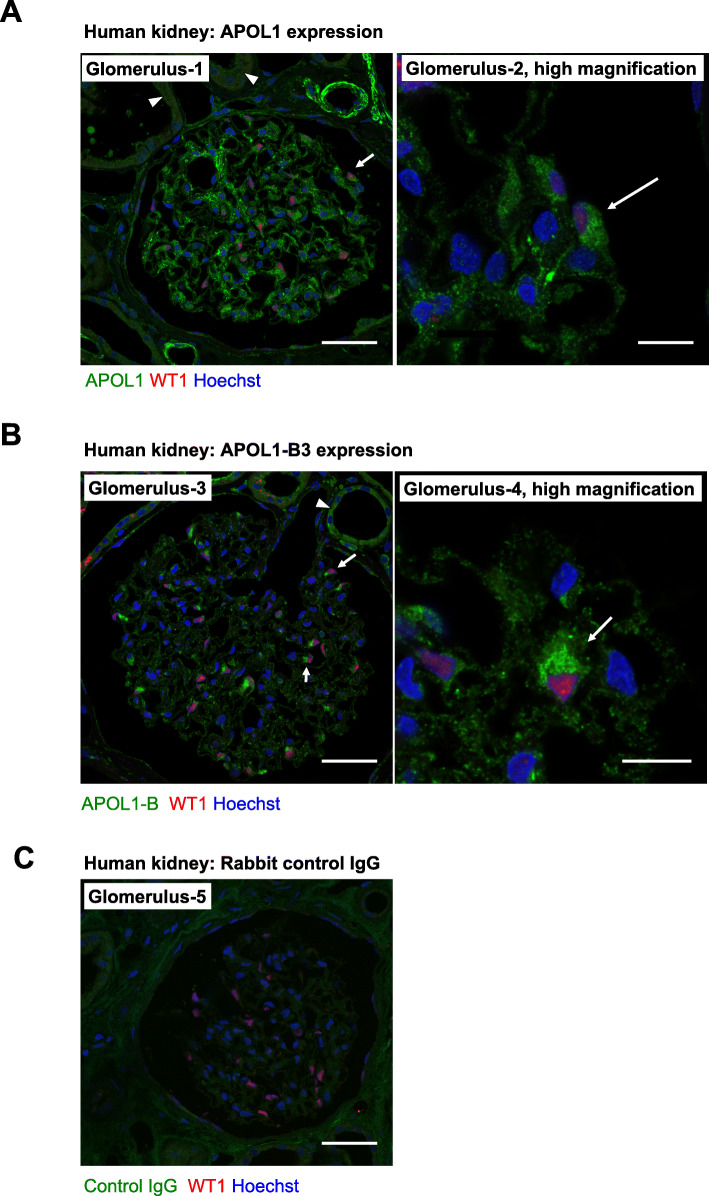
Human kidney cells expressed APOL1-B3 in vivo. **a**-**c** To examine the presence of APOL1 isoforms in human kidney, human autopsy kidney specimens were used for immunohistochemical analysis using APOL1-B antibody and rabbit monoclonal antibody (RPR2907). Podocytes expressed APOL1 as shown by staining with RPR2907 antibody (**a**), and with APOL1-B antibody recognizing APOL1-B3 (**b**). Control rabbit IgG did not stain podocytes (**c**). Wilms tumor1 (WT1) was used as a podocyte marker. All glomeruli were derived from identical kidney. Scale bar indicates 40 μm in regular and 10 μm in high magnification images. Arrow and arrowhead indicate podocytes and tubular cell respectively

Next, we examined the localization of APOL1-B3 in kidney tissue. We first selected wild-type mouse and BAC/APOL1 transgenic mouse kidneys for these studies, since mice do not express APOL1 and thus wild-type mice can be used as a negative control. BAC/APOL1-G0 mouse kidneys, which expressed V2–3 mRNA, were subjected to immunohistochemical analysis using the rabbit polyclonal APOL1-B antibody and the rabbit anti-APOL1 monoclonal antibody (clone EPR2907). In BAC/APOL1 mouse kidney, we found APOL1 immunoreactive signal in podocytes and tubular cells using the monoclonal APOL1 antibody (EPR2907) (Supplementary Figure 2D), and also with the polyclonal APOL1-B antibody (Supplementary Figure 2E), suggesting that both podocytes and tubular cells expressed APOL1-B3. These antibodies did not stain glomeruli in wild-type mouse kidney (Fig. 2d, e). The polyclonal APOL1-B antibody produced background staining of tubular cells in both wild type and transgenic mice. Next, we examined the expression of APOL1 in autopsy samples from human kidneys. Similar to the pattern seen in the BAC/APOL1 mouse kidney, in human kidney both the monoclonal APOL1 antibody (EPR2907) (Fig. 2a) and the polyclonal APOL1-B antibody (Fig. 2b) stained the cytoplasm of podocyte and tubular cells, while the antibody against the podocyte marker WT1 stained podocyte nuclei. Rabbit IgG was used as negative control (Fig. 2c). The monoclonal APOL1 antibody (EPR2907) stained vascular wall in the autopsy kidney. In summary, our results gathered from kidneys of transgenic mice and humans indicated that podocyte and tubular cells expressed APOL1-B3 in vivo.

### APOL1 mRNA expression was enhanced by IL-1β

**Fig. 3:**
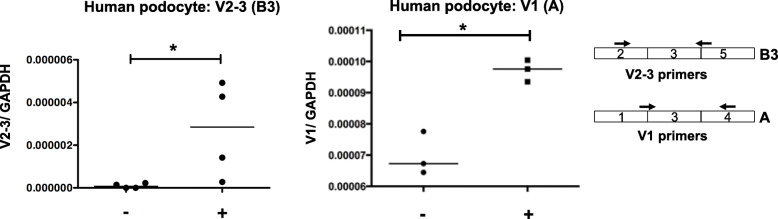
APOL1-B3 regulated proinflammatory signaling in vitro. Immortalized human podocytes were stimulated by IL-1β (15 ng/ ml, 3 h). The expression of the APOL1 splice variant was examined by qPCR, using V2–3 primers and V1 primers as indicated by arrows in the above exon schema. The graph shows the relative expression of IL-1β to GAPDH. (Student’s t test, *p* < 0.05)

It has been shown that APOL1 mRNA expression increases upon proinflammatory stimulation [15, 21, 22]. We examined the expression of APOL1 mRNA variants in human podocytes that were differentiated for three days quantitatively, using V2–3 and V1 specific primers. We observed that expression of both V2–3 and V1 variants was increased following IL-1β stimulation (Fig. 3). This was consistent with a previous report [15, 21, 22]. However, the role of APOL1 in the regulation of inflammatory signaling is unknown.

### CAG/APOL1-B3 transgenic mice expressed APOL1-B3 in podocytes and tubular cells

**Fig. 4:**
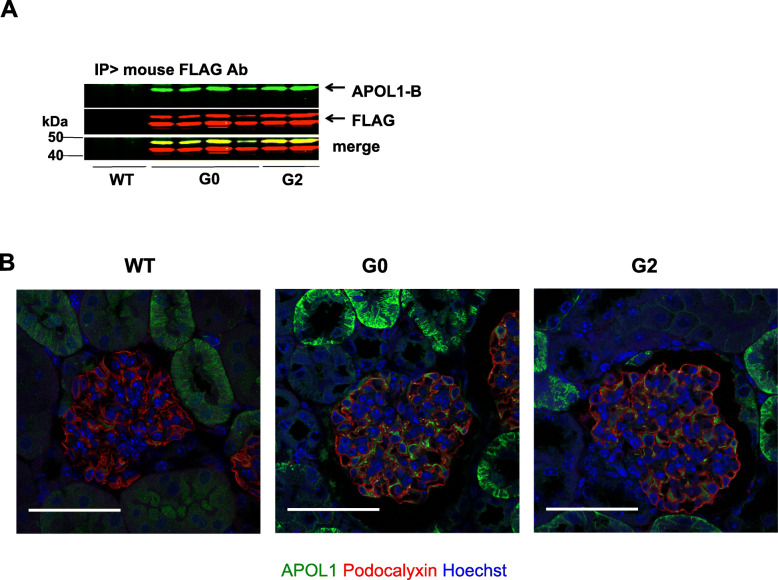
CAG-APOL1-B3 transgenic mice expressed APOL1-B3 in podocytes and tubular cells. **a** CAG (chicken ß-actin promoter) APOL1-B3-FLAG transgenic mice were generated in a FVB/N mouse background. To confirm the expression of APOL1-B3 in the kidney, extracts from whole kidneys were subjected to Western analysis using indicated antibodies, showing that APOL1-B3 was expressed. **b** Localization of APOL1-B3 in the kidney was determined by immunohistochemistry using rabbit monoclonal antibody (EPR2907). Podocalyxin was used as a podocyte marker. Scale bar shows 40 μm

Following the results shown in Fig. 3, we asked whether the APOL1-B3 renal risk variant induces podocyte damage by promoting inflammatory stress in vivo. For this purpose, we generated CAG-APOL1-B3 transgenic mice, which expressed FLAG-tagged APOL1-B3-G0 or APOL1-B3-G2. Expression of APOL1-B3 protein in the mouse kidney was confirmed by western analysis. APOL1-B3 protein from whole kidney was immunoprecipitated with anti-FLAG antibody and characterized using APOL1-B and anti-FLAG antibodies, showing that APOL1-B3 was expressed in the transgenic mouse kidney (Fig. 4a). In the immunoblot, the lower FLAG signal band corresponded to truncated APOL1-B3 as discussed above.

We examined the cellular localization of APOL1-B3 in mouse kidney sections by immunohistochemistry using rabbit anti-APOL1 monoclonal antibody (clone: EPR2907). Immunoreactive signal was present in the podocytes and tubular cells of APOL1-B3-G0 and -G2 expressing mice (Fig. 4b). There were no remarkable pathological findings in the kidneys from 8-week-old APOL1-B3 transgenic mice as judged by light microscopy (Supplementary Figure 3).

### APOL1-B3-G2 transgenic mice manifested enhanced albuminuria and IL-1β production following uninephrectomy

**Fig. 5:**
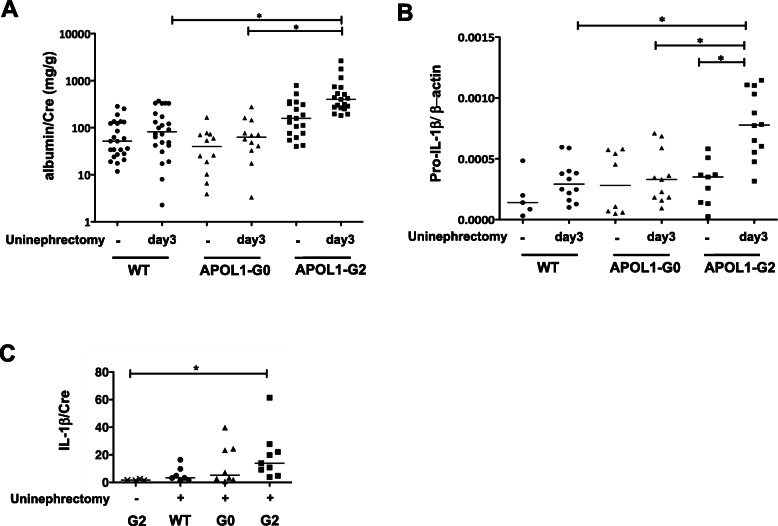
APOL1-B3-G2 enhanced podocyte damage and IL-1β production in vivo. **a** Three days after uninephrectomy, the urine ACR was increased in APOL1-B3-G2 mice compared to wild-type mice and APOL1-B3-G0 mice. **b** Following uninephrectomy, pro-IL-1β mRNA from isolated glomeruli was upregulated in APOL1-B3-G2 but not in APOL1-B3-G0 mice. Data shown as expression of pro-IL-1β relative to β-actin. **c** IL-1β levels in the urine, standardized by urine creatinine (Cr) concentration, were found to be significantly higher in APOL1-B3-G2 transgenic mice compared to baseline. Data shown as IL-1β (pg/ml)/Cr (mg/dl). Bars are shown median, asterisk shows *p* < 0.05

We hypothesized that APOL1-B3-G2 might be involved in podocyte damage. We evaluated podocyte damage and glomerular pro-IL-1β mRNA level in APOL1-B3 mice after uninephrectomy. Three days following uninephrectomy, ACR was significantly higher in APOL1-B3-G2 mice compared to wild-type and APOL1-B3-G0 mice (Fig. 5a). To elucidate mechanisms of podocyte injury, we isolated glomeruli from the remnant kidney and bone marrow cells as a positive control. Glomerular APOL1 mRNA levels did not differ among mouse lines (Supplementary Figure 4A). Isolated glomeruli expressed nephrin that was, as expected, absent in isolated bone marrow cells, and both glomerular-[add] cells and bone marrow cells expressed TLR4 mRNA (Supplementary Figure 4B). TLR4 signaling has been associated with proteinuria [23], and podocyte TLR4 signaling contributes to podocyte injury in experimental animal models [24]. Further, podocyte NF-κB pathway activation has been associated with proteinuria in a glomerulonephritis model [25, 26]. Glomerular expression of TLR4 mRNA was significantly upregulated in APOL1-B3-G2 mice following uninephrectomy (Supplementary Figure 4C) and pro-IL-1β mRNA levels were higher at day 3 in APOL1-B3-G2 mice than in the other groups (Fig. 5b). Moreover, IL-1β in the urine was increased following uninephrectomy, and the change reached significance in APOL1-B3-G2 mice, suggesting enhancement of IL-1β processing in the kidney (Fig. 5c). These data indicated that the uninephrectomy was associated with induction of TLR4 signaling, pro-IL-1β mRNA expression, and IL-1β production in the remnant kidney, possibly involving an activation of the inflammasome.

We further investigated the effects of APOL1-B3-G0 and APOL1-B3-G2 expression on podocytes in kidney sections of mice prior to and following uninephrectomy. We quantitated podocyte numbers with p57 staining and determined p57 staining intensity as a surrogate for podocyte injury (Supplementary Figure 4D - I). We observed that the number of podocytes differed little when comparing pre- and post-uninephrectomy kidney samples for wild-type (FVB) mice and APOL-B3-G0 mice (Supplementary Figure 4E). In contrast, for the risk variant APOL1-B3-G2 mice, podocyte number decreased by ~ 15% following nephrectomy, a difference which reached statistical significance (*p* = 0.022). We next measured glomerular areas (Supplementary Figure 4F). In APOL1-B3 variant samples, glomerular areas changed little and without reaching statistical significance. In contrast, in wild-type samples, there was a 14% increase in glomerular area (*p* = 0.04). However, the ratio of the podocyte number per glomerular area did not change significantly for any group (Supplementary Figure 4G). Finally, we investigated p57 staining intensities as a surrogate for podocyte-specific changes in cellular status, such as observed during podocyte injury ( [27] (Supplementary Figure 4H - I). When comparing pre- and post-uninephrectomy samples, mean p57 staining intensities declined both per glomerular area (~ 32%) and per podocyte (~ 16%) only for the APOL1-B3-G2 risk variant (*p* < 0.0001). Differences were not significant for p57 staining intensities when calculated ‘per glomerulus’ for wild-type and APOL1-B3-G0 mice. However, p57 intensity values increased for both wild-type and APOL1-B3-G0 mice (~ 8 and 6%, respectively) significantly when evaluated ‘per podocyte’ (*p* = 0.0001 and *p* = 0.013, respectively). This observation that podocyte numbers and podocyte p57 expression were lower in APOL1-B3-G2 mice following uninephrectomy is consistent with the presence of greater microalbuminuria, suggesting increased podocyte injury. In summary, APOL1-B3-G2 expression aggravated podocyte damage and enhanced the proinflammatory response in glomerular cells after uninephrectomy.

### APOL1-B3 associated with NLRP12

**Fig. 6:**
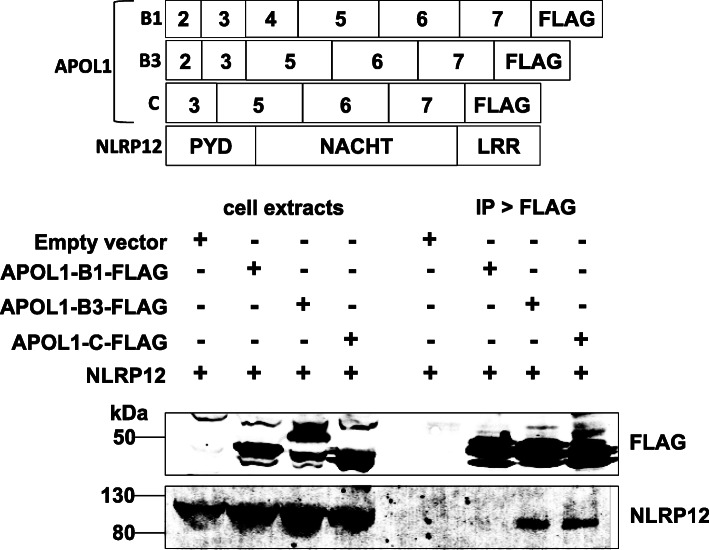
APOL1-B3 and C isoforms uniquely interacted with NLRP12. Shown are the structure of FLAG-tagged APOL1 isoforms expression vectors. Also shown is the design of the NLRP12 expression vector highlighting the three major domains (pyrin domain, PYD; NACHT domain, and leucine rich repeat, LRR) of this protein. The constructs were transiently transfected into HeLa cells. APOL1-B3 and APOL1-C isoforms co-immunoprecipitated with NLRP12, while the secreted APOL1-B1 isoform did not. APOL1-B3 interacted with NLRP12 but not APOL1-B1, which is identical to APOL1-A in its amino acid sequence

We wished to determine whether APOL1-B3 modulates proinflammatory signaling via either the IL-1 receptor or TLR4. We investigated APOL1-B3 binding partners by using mass spectrometry analysis of proteins that coprecipitated with APOL1-B3-FLAG from APOL1-B3-FLAG stably transfected HeLa cells. We identified a 12-amino acid sequence (TVVMQGAAGIGK) specific to NLRP12, suggesting an interaction between APOL1-B3 and NLRP12. To identify APOL1 isoforms that bind NLRP12, we cotransfected NLRP12 and FLAG-tagged APOL1 isoforms into HeLa cells, and immunoprecipitated APOL1 with anti-FLAG antibodies. Transfected NLRP12 was coprecipitated with APOL1-B3-FLAG and APOL1-C-FLAG but not with APOL1-B1-FLAG (Fig. 6) or APOL1-A-FLAG (Supplementary Figure 5A). These data indicated that NLRP12 specifically associated with the predicted intracellular forms of APOL1, namely B3 and C, but not the secreted forms of APOL1, namely B1 or A, in concordance with the notion that presence or absence of a signal peptide determines the localization of APOL1 isoforms and hence their cellular interactions.

Next, we examined the interaction of APOL1-B3 renal risk variants with NLRP12. All wild-type and renal risk variants of APOL1-B3 associated with NLRP12 as shown in pull-down experiments (Supplementary Figure 5B). However, the reason for the differences in the observed degree of co-precipitation of NLRP12 by the APOL1 variants remains to be determined.

Finally, we examined which domains of APOL1-B3 were responsible for the interaction with NLRP12. We made APOL1-B3-G0 constructs in which the C-terminal SRA binding domain (C-del) or the membrane-addressing domain (M-del) was deleted. NLRP12 was co-precipitated with full-length APOL1 and both domain deleted APOL1-B3 polypeptides; the C-terminal domain seemed to be dispensable for association with NLRP12 (Supplementary Figure 5C). It is not clear whether APOL1-B3 and NLRP12 interacted directly. It remains to be shown how renal risk variants located in the C-terminal affect binding to and to inhibition of NLRP12.

### APOL1-B3 regulated NLRP12 function

We addressed the consequences of APOL1-B3 association with NLRP12 in HeLa cells stably transfected with empty vector, APOL1-B3-G0 or APOL1-B3-G2 constructs. We examined the effect of APOL1-B3-enhanced IL-1 receptor signaling in the setting of NLRP12 overexpression achieved by transient transfection of HeLa cells. Compared to control HeLa cells, APOL1-B3 transfected cells (G0 and G2) both showed enhanced ERK phosphorylation at 5 min after stimulation with IL-1β. Compared to empty vector, NLRP12 overexpression downregulated phosphorylation of ERK in each group (empty vector, APOL1-B3-G0, and APOL1-B3-G2 stably transfected-HeLa cells), as expected for a negative regulator of the immune process (Supplementary Figure 6). These data indicated that exogenous transfected NLRP12 attenuated IL-1 receptor signaling and that APOL1-B3 (G0 and G2) did not overcome this inhibitory function at supraphysiologic levels of NLRP12. In these data, the effect of over-expressed APOL1-B3 on signaling was similar for the G0 and G2 variants in HeLa cells.

Our data suggest that overexpressed APOL1-B3 (G0 and G2) functionally antagonized endogenous NLRP12 in the early phase of IL-1 receptor activation in vitro. How APOL1-B3-G2 and NLRP12 are functionally linked in the kidney remains unclear, as we did not investigate the consequences of overexpression of NLRP12 in the kidney of APOL1-B3 transgenic mice.

## Discussion

We report an APOL1 isoform, APOL1-B3, that regulates proinflammatory signaling, and associates with NLRP12, a Toll-like receptor signaling regulator. As previously reported, APOL1 has multiple mRNA splice variants [16] and we confirmed the expression of these variants in immortalized human podocytes and in human and BAC/APOL1 mouse kidney. We identified a novel mRNA variant, APOL1-V2–3, that lacks the signal sequence, which suggests that APOL1-B3 is a cytoplasmic protein and not secreted as is the case for the APOL1-A isoform. Khatua et al. reported that immortalized human podocytes express APOL1 mRNA, which include splice variants that lack exon 4 and which correspond to APOL1-B3 and APOL1-C [22].

Kidney transplant studies suggest that kidney injury is the result of kidney-expressed APOL1 and not that of circulating APOL1 [3, 4]. Kidney-expressed APOL1 mRNA V1 (A), V2–1 (B1), V2–3 (B3) and V4 (C) are predicted to produce APOL1 isoform A, B3 and C. Prior reports [28, 29] on podocyte APOL1 expression have used antibodies that were not specific for individual APOL1 splice isoforms (A, B3, and C). Using an APOL1-B3 specific antibody, we showed that APOL1-B3 is present in human podocytes human and BAC/APOL1 mouse kidney.

We demonstrated that transgenic APOL1-B3-G2 expression enhanced proinflammatory response in the glomeruli and albuminuria following uninephrectomy. Not only in APOL1-B3-G2 glomeruli but also in wild type and APOL1-B3-G0 mice glomeruli, TLR4 mRNA was numerically up-regulated after uninephrectomy, but pro-IL-1β mRNA was enhanced only in APOL1-B3-G2. This suggest that APOL1-B3-G2 might interfere with negative regulation of TLR4 signaling. The resulting prolonged TLR4 signaling and cellular inflammation would promote progressive podocyte dysfunction and fibrosis. This agrees with the observation that APOL1 gene variants are associated with glomerular disease, which tends to manifest in a more rapid progression to end-stage kidney disease [30]. TLR4 signaling has been implicated in the pathogenesis of glomerular disease, including cryoglobulinemia in mice [24] and diabetic nephropathy in humans [31]. Data from the NEPTUNE study of glomerular disease supports a role for APOL1 variants in enhancing inflammatory signaling. Thus, two of the three glomerular transcripts that are upregulated in APOL1 high-risk genotype subjects compared to low-risk subjects are the proinflammatory chemokines CXCL9 and CXCL11 [6], which are enhanced by TNF, IL-1 and TLR signaling [32, 33].

It is well-established that nephrectomy activates the renin-angiotensin system. In addition, studies have shown that angiotensin II induces TLR4 expression [34–36]. Here, we showed that glomerular pro-IL-1β mRNA and urine IL-1β levels were increased in APOL1-B3-G2 mice. APOL1-B3-G2 promoted podocyte damage following uninephrectomy, possibly by increasing glomerular synthesis and processing of pro-IL-1β. However, we have not evaluated the contribution of podocyte-derived IL-1β in podocyte damage in this setting.

In this study, constitutively-expressed transgenic APOL1-B3 did not induce glomerulosclerosis at steady-state. However, doxycycline-induced renal risk-APOL1 over-expression in podocytes caused severe podocyte damage, glomerulosclerosis, and resulted in enhanced expression of CXC chemokines in the kidney, similar to that reported in the NEPTUNE clinical study [6, 37]. Thus, APOL1 renal risk variant-enhanced inflammatory processes are involved in the pathogenesis of nephropathy.

Using a proteomics approach, we established that APOL1-B3 associates with NLRP12. NRLP12 is a negative regulator of TLR signaling. As predicted, NLRP12 over-expression inhibited APOL1-B3-mediated enhancement of IL-1 receptor signaling. This indicated that APOL1-B3 antagonized NLRP12, thereby amplifying IL-1 receptor signaling. We showed that the cytoplasmic protein NLRP12 interacted with isoforms APOL1-B3 and APOL1-C, but not with isoform APOL1-A. This evidence supports the idea that cytoplasmic APOL1 isoforms, but not the secreted APOL1-A isoform, contribute to intracellular proinflammatory signaling. Thus, APOL1 isoforms make distinct contributions to APOL1 pathogenesis.

The mechanism by which APOL1-B3 enhances inflammatory signaling appears to be, at least in part, by regulation of NLRP12 function. NLRP12 inhibits early events in TLR signaling by preventing hyperphosphorylation of IRAK-1 in vitro [10]. APOL1-B3, both wild-type (G0) and renal risk variant (G2), enhanced the early events of IL-1 receptor signaling, the phosphorylation of MAPK and ERK in APOL1-B3-HeLa cells. Although NLRP12 coprecipitated with wild-type and renal risk variants of APOL1-B3, the molecular mechanism of how the renal risk variant APOL1-B3-G2 enhances proinflammatory signaling associated with NLRP12 remains to be shown. APOL1-B3-G2 but not APOL1-B3-G0 enhanced pro-IL-1β mRNA expression in vivo (transgenic mice) but this risk-variant dependent signaling was not observed when investigating the effect of APOL1-B3-G0 and APOL1-B3-G2 on NLRP12 in IL-1 receptor signaling in vitro (cultured HeLa cells). This difference might be due to the presence of multiple targets of NLRP12 in proinflammatory signaling involving TLRs, IL-1 receptor and non-canonical NF-κB pathways in various cell types in vivo, or due to the supra-physiological effect of high-dose of IL-1β or over-expression of APOL1 in vitro.

Our data support and extend prior work that APOL1 risk variants induce inflammatory stress and cause cellular injury, as has been shown using cultured cells and/or transgenic mouse or human tissues. For example, recent studies have shown that enhanced K^+^ efflux in an APOL1 risk milieu promoted the development of inflammatory stress [38]. Other studies have shown that APOL1 variant expression impairs autophagy, increases endoplasmic reticulum stress, and induces mitochondrial dysfunction [39]. Here, we have shown that the APOL1-B3 isoform is sufficient to promote inflammatory stress. This does not exclude, however, a role for other splice isoforms in this process.

In conclusion, we propose that the APOL1-B3-G2 risk variant promotes inflammatory signaling in podocytes, leading to glomerular damage. This pathway may represent a novel therapeutic target for APOL1 nephropathy.

## Supplementary information

**Additional file 1: Figure S1.** Kidney tissue expresses APOL1 splice variant V2–3. (A) The expression of the V2–3 splice variant was detected in human kidney and lung. APOL1 V2–3 primer specifically amplified the V2–3 splice variant. The V2–3 reverse primer bridges the exon 3 and exon 5 junction as shown by arrows in the above exon schema. (B) The left panel shows APOL1 V1 variant expression in the kidneys of BAC-APOL1-G0 transgenic mice. V1 plasmid served as positive control and actin primers demonstrated intact cDNA. In the right panel, BAC-APOL1-G0 mouse kidney also expresses V2–1 and V2–3, with positive plasmid controls shown. Thus BAC-APOL1-G0 mice express three APOL1 splicing variants: V1, V2–1 and V2–3 (isoform: A, B1 and B3). (C) APOL1 splice variants were cloned from BAC-APOL1 mouse kidney and human kidney using TAcloning. We submitted the V2–3 mRNA sequence (human podocyte) to NCBI GenBank (#KX192151). **Figure S2.** APOL1-B3 was specifically detected by APOL1-B antibody, and was not present in human serum. (A) APOL1-B antibody was affinity purified from serum obtained from rabbit immunized with APOL1-B isoform-specific peptide (epitopes are shown in exons 2 and 3). Extracts from human podocytes transfected with FLAG-tagged APOL1-B1, −B2 and -B3 constructs were immunoprecipitated with anti-FLAG antibody, followed by western analysis using both APOL1-B antibody, and mouse anti-APOL1 C-terminal antibody (Sigma: clone CL0171). To characterize antibody specificity, the antibody was preincubated with immunizing peptide at molar ratios (antibody:antigen 1:40). No signal was observed in any lanes, showing that APOL1-B antibody was specific for the antigen (top panel). The isoform specificity of APOL1-B antibody was determined. Mouse APOL1 C-terminal antibody (CL0171) recognized all three isoforms, but the APOL1-B antibody was specific for the B3 isoform. (B, C) To identify APOL1 isoforms in human serum, serum samples were subjected to western analysis using APOL1-B and C-terminal APOL1 antibodies (Sigma; rabbit polyclonal antibody). Human serum did not contain APOL1-B3 (B) but, instead, the circulating form of APOL1, isoform A, was detected by the APOL1 C-terminal antibody (C). Transfected APOL1-B3 and APOL1-A were used as positive controls. (D, E) To examine the localization of APOL1 isoforms in kidney, BAC APOL1-G1 mouse kidney was used for immunohistochemical analysis using rabbit monoclonal APOL1 antibody (Abcam: clone RPR2907) (D), and the polyclonal APOL1-B antibody (E). Podocalyxin was used as a podocyte marker. Wild-type mice, left panels in D and E, showed no staining, as expected. Podocytes expressed APOL1 as identified by staining with rabbit APOL1 monoclonal antibody, and with APOL1-B antibody recognizing APOL1-B3. Thus podocytes express APOL1-B3, although other isoforms may also be present. Scale bar shows 20 μm. Arrow and arrowhead indicated podocytes and tubular cells, respectively. **Figure S3.** PAS staining of kidney of transgenic mouse showed normal appearance. PAS staining of kidney sections of 8-weeks-old APOL1-B3 transgenic mice showed no remarkable histological changes in the kidney. Scale bar indicates 40 μm. **Figure S4.** TLR4 was upregulated in the remnant kidney after uninephrectomy. (A) Expression of APOL1 mRNA from isolate glomeruli was similar in APOL1-B3-G0 and –G2 mice after uninephrectomy. G0: *N* = 6, G2: *N* = 9. APOL1 expression is shown relative to actin. (B) Following uninephrectomy, nephrin and TLR4 were expressed in glomeruli isolated from CAG-APOL1-B3 mice. Bone marrow cDNA was used as a positive control for TLR4 and as a negative control for nephrin. (C) Glomerular TRL4 mRNA was upregulated following uninephrectomy in all groups and reached statistical significance compared to baseline in APOL1-B3-G2 transgenic mice. (D) Immunohistochemistry of formalin-fixed, paraffin-embedded kidney cortex section from wild-type (FVB), APOL1-B3-G0, and APOL1- B3-G2 mice before and after uninephrectomy showing staining for p57 of (from left to right, top row: pre-uninephrectomy; bottom row: post-uninephrectomy): wild-type, APOL1-B3-G0, and APOL1-B3-G2. Quantitative analysis showing, for each group, (E) the number of podocytes, (F) area of glomeruli, (G) ratio of podocyte number over glomerular area, (H) p57 staining intensities per glomerular area, and (I) p57 staining intensities per podocytes. (ns, not significant; * < 0.03322; **** < 0.0001) APOL1-FLAG. **Figure S5.** Renal risk variant APOL1 associated with NLRP12. (A) The schematic depicts the exon composition of the APOL1-FLAG expression constructs and the domain architecture of NLRP12, respectively. Pull-down experiments of APOL1-NLRP12 cotransfected HeLa cells with anti-FLAG antibodies showed in immunoblots that APOL1-B3 but not APOL1-A interacted with NLRP12. (B) NLRP12 and FLAG-tagged APOL1-B3 variants (G0, G1, G2) were transfected into HeLa cells, followed by immunoprecipitation with anti-FLAG antibody and western analysis for NLRP12. (C) To further characterize the interactions with APOL1-B3 with NLRP12, we showed similarly that deletion of the C-terminal domain (C-del) and the membrane-addressing domain of APOL1-B3 interacted with NLRP12. APOL1-B3 C-terminal domain was not required in the interaction with NLRP12. **Figure S6.** Overexpression of NLRP12 antagonized APOL1-B3. Compared to vector (V) control, overexpression of NLRP12 (N) suppressed phospho-ERK induced by exposure to IL-1b (15 ng/ml) for 5 or 30 min in the presence of APOL1-B3-G0 and APOL1-B3- G2. The expression levels of FLAG-APOL1-B3 protein were similar between groups, confirming equivalent transfection efficiencies. This experiment was repeated twice with reproducible results. For the western blot image, protein was loaded on two different gels and then transferred to a single membrane. The resulting image was trimmed and is shows as one panel.

**Additional file 2. **Supplementary methods. **Table S1.** Antibodies and nuclear staining. **Table S2.** Primers used for cloning APOL1 splice variants. **Table S3.** Primers use for RT-PCR, qPCR and genotyping. **Table S4.** Primers used for sub-cloning of APOL1-FLAG expression vector. **Table S5.** Primers used for sub-cloning of CAG-APOL1-B3 expression vector. **Table S6.** Primers used for mutagenesis of APOL1-B3 FLAG vectors. **Table S7.** Primers used to generate NRLP12 expression vectors. **Table S8.** Number of mice used in Fig. 5.

## Data Availability

Data are available from JBK (author). Transgenic mice may be available in collaboration with Merck, Inc.

## Abbreviations

APOL1: Apolipoprotein L1
BAC: Bacterial artificial chromosome
IRAK1: Interleukin-1 receptor associated protein kinase
NEPTUNE: Nephrotic Diseases Study Network
NOD: Nucleotide-binding oligomerization domain-like
NLRP: Nucleotide-binding oligomerization domain, leucine rich repeat and pyrin domain containing
TLR: Toll-like receptor
