# Supplementary material for: APOL1 renal risk variants exacerbate podocyte injury by increasing inflammatory stress
Source: BMC Nephrol. 2020 Aug 27;21:371. doi: 10.1186/s12882-020-01995-3 (PMC7450955; doi:10.1186/s12882-020-01995-3)
Supplement: Supplementary file 1 — Additional file 1: Figure S1. Kidney tissue expresses APOL1 splice variant V2–3. (A) The expression of the V2–3 splice variant was detected in human kidney and lung. APOL1 V2–3 primer specifically amplified the V2–3 splice variant. The V2–3 reverse primer bridges the exon 3 and exon 5 junction as shown by arrows in the above exon schema. (B) The left panel shows APOL1 V1 variant expression in the kidneys of BAC-APOL1-G0 transgenic mice. V1 plasmid served as positive control and actin primers demonstrated intact cDNA. In the right panel, BAC-APOL1-G0 mouse kidney also expresses V2–1 and V2–3, with positive plasmid controls shown. Thus BAC-APOL1-G0 mice express three APOL1 splicing variants: V1, V2–1 and V2–3 (isoform: A, B1 and B3). (C) APOL1 splice variants were cloned from BAC-APOL1 mouse kidney and human kidney using TAcloning. We submitted the V2–3 mRNA sequence (human podocyte) to NCBI GenBank (#KX192151). Figure S2. APOL1-B3 was specifically detected by APOL1-B antibody, and was not present in human serum. (A) APOL1-B antibody was affinity purified from serum obtained from rabbit immunized with APOL1-B isoform-specific peptide (epitopes are shown in exons 2 and 3). Extracts from human podocytes transfected with FLAG-tagged APOL1-B1, −B2 and -B3 constructs were immunoprecipitated with anti-FLAG antibody, followed by western analysis using both APOL1-B antibody, and mouse anti-APOL1 C-terminal antibody (Sigma: clone CL0171). To characterize antibody specificity, the antibody was preincubated with immunizing peptide at molar ratios (antibody:antigen 1:40). No signal was observed in any lanes, showing that APOL1-B antibody was specific for the antigen (top panel). The isoform specificity of APOL1-B antibody was determined. Mouse APOL1 C-terminal antibody (CL0171) recognized all three isoforms, but the APOL1-B antibody was specific for the B3 isoform. (B, C) To identify APOL1 isoforms in human serum, serum samples were subjected to western analysis using APOL1-B and [file 12882_2020_1995_MOESM1_ESM.pdf]

Supplementary Figure 1.

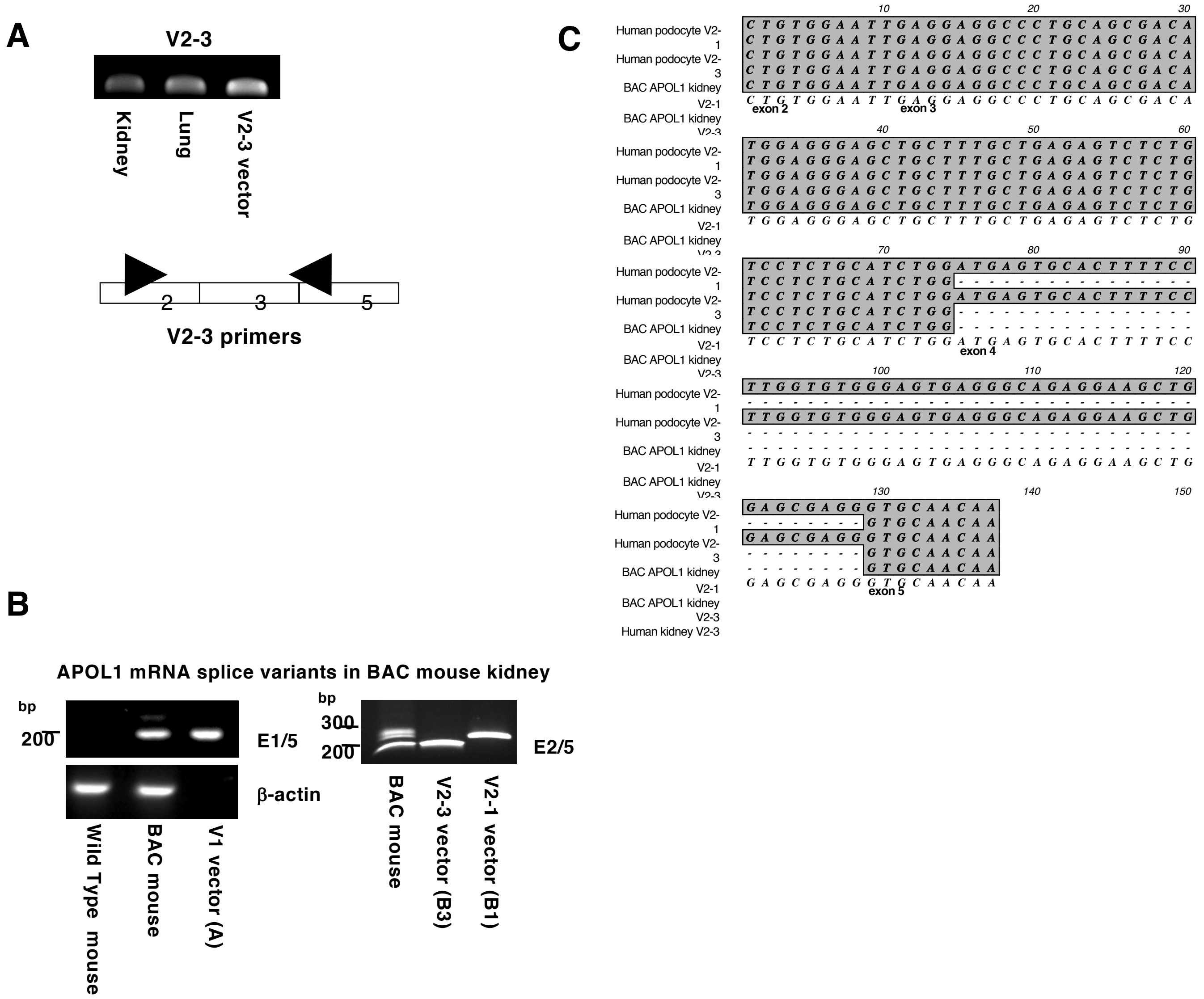

Supplementary Figure 1. Kidney tissue expresses APOL1 splice variant V2-3 .
(**A**) The expression of the V2-3 splice variant was detected in human kidney and lung. APOL1 V2-3 primer specifically amplified the V2-3 splice variant. The V2-3 reverse primer bridges the exon 3 and exon 5 junction as shown by arrows in the above exon schema.
(**B**) The left panel shows APOL1 V1 variant expression in the kidneys of BAC-APOL1-G0 transgenic mice. V1 plasmid served as positive control and actin primers demonstrated intact cDNA. In the right panel, BAC-APOL1-G0 mouse kidney also expresses V2-1 and V2-3, with positive plasmid controls shown. Thus BAC-APOL1-G0 mice express three APOL1 splicing variants: V1, V2-1 and V2-3 (isoform: A, B1 and B3).
(**C**) APOL1 splice variants were cloned from BAC-APOL1 mouse kidney and human kidney using TA-cloning. We submitted the V2-3 mRNA sequence (human podocyte) to NCBI GenBank (#KX192151).

Supplementary Figure 2.

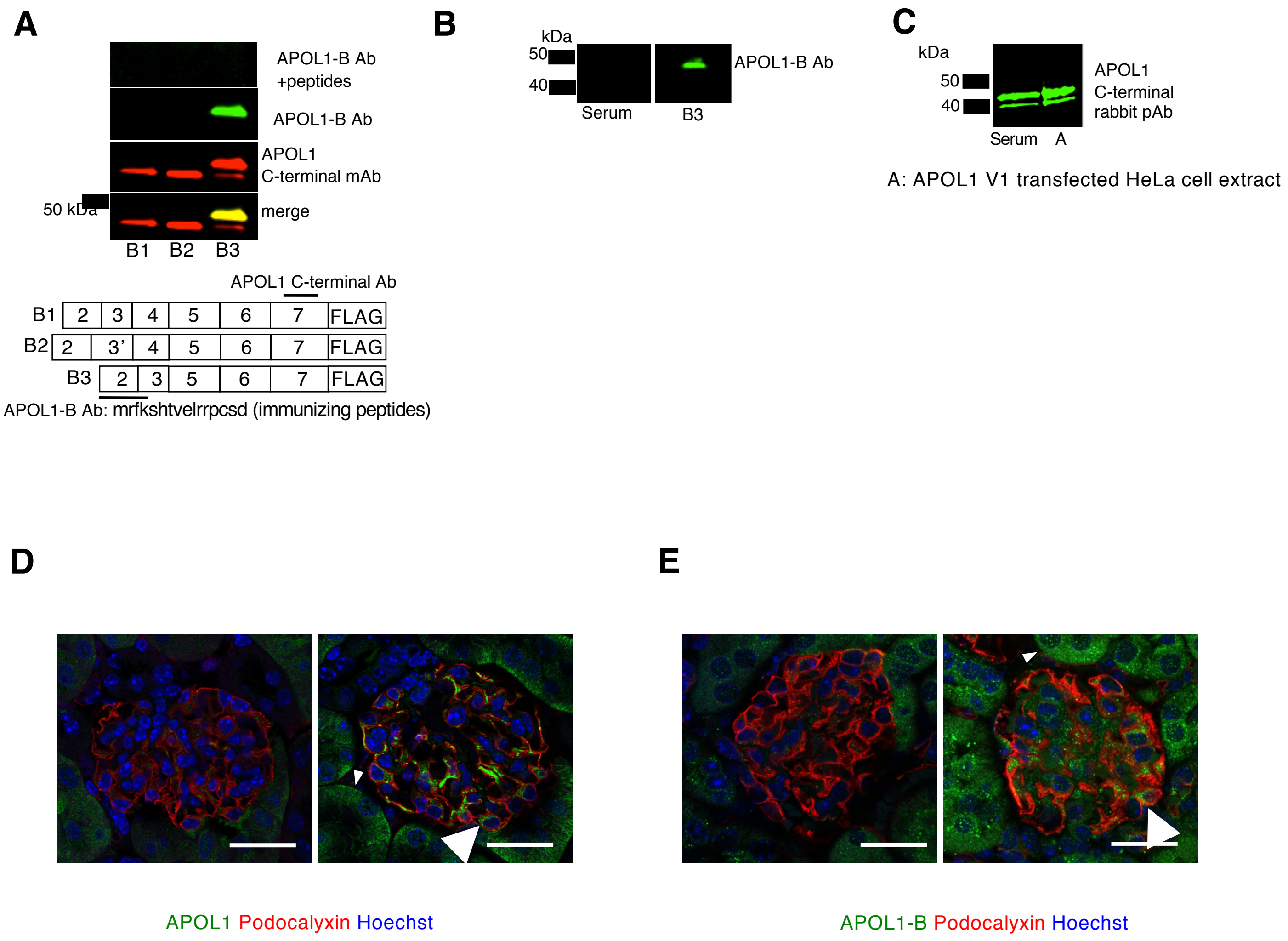

Supplementary Figure 2. APOL1-B3 was specifically detected by APOL1-B antibody, and was not present in human serum.
(**A**) APOL1-B antibody was affinity purified from serum obtained from rabbit immunized with APOL1-B isoform-specific peptide (epitopes are shown in exons 2 and 3). Extracts from human podocytes transfected with FLAG-tagged APOL1-B1, -B2 and -B3 constructs were immunoprecipitated with anti-FLAG antibody, followed by western analysis using both APOL1-B antibody, and mouse anti-APOL1 C-terminal antibody (Sigma: clone CL0171). To characterize antibody specificity, the antibody was pre-incubated with immunizing peptide at molar ratios (antibody:antigen 1:40). No signal was observed in any lanes, showing that APOL1-B antibody was specific for the antigen (top panel). The isoform specificity of APOL1-B antibody was determined. Mouse APOL1 C-terminal antibody (CL0171) recognized all three isoforms, but the APOL1-B antibody was specific for the B3 isoform. (**B, C**) To identify APOL1 isoforms in human serum, serum samples were subjected to western analysis using APOL1-B and C-terminal APOL1 antibodies (Sigma; rabbit polyclonal antibody). Human serum did not contain APOL1-B3 (**B**) but, instead, the circulating form of APOL1, isoform A, was detected by the APOL1 C-terminal antibody (**C**). Transfected APOL1-B3 and APOL1-A were used as positive controls. (**D, E**) To examine the localization of APOL1 isoforms in kidney, BAC APOL1-G1 mouse kidney was used for immunohistochemical analysis using rabbit monoclonal APOL1 antibody (Abcam: clone RPR2907) (**D)**, and the polyclonal APOL1-B antibody (**E)**. Podocalyxin was used as a podocyte marker. Wild-type mice, left panels in D and E, showed no staining, as expected. Podocytes expressed APOL1 as identified by staining with rabbit APOL1 monoclonal antibody, and with APOL1-B antibody recognizing APOL1-B3. Thus podocytes express APOL1-B3, although other isoforms may also be present. Scale bar shows 20 μm. Arrow and arrowhead indicated podocytes and tubular cells, respectively.

Supplementary Figure 3.

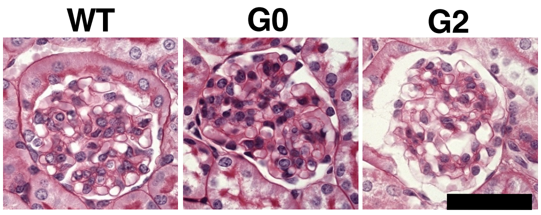

Supplementary Figure 3. PAS staining of kidney of transgenic mouse showed normal appearance. PAS staining of kidney sections of 8-weeks-old APOL1-B3 transgenic mice showed no remarkable histological changes in the kidney. Scale bar indicates 40 μm.

Supplementary Figure 4.

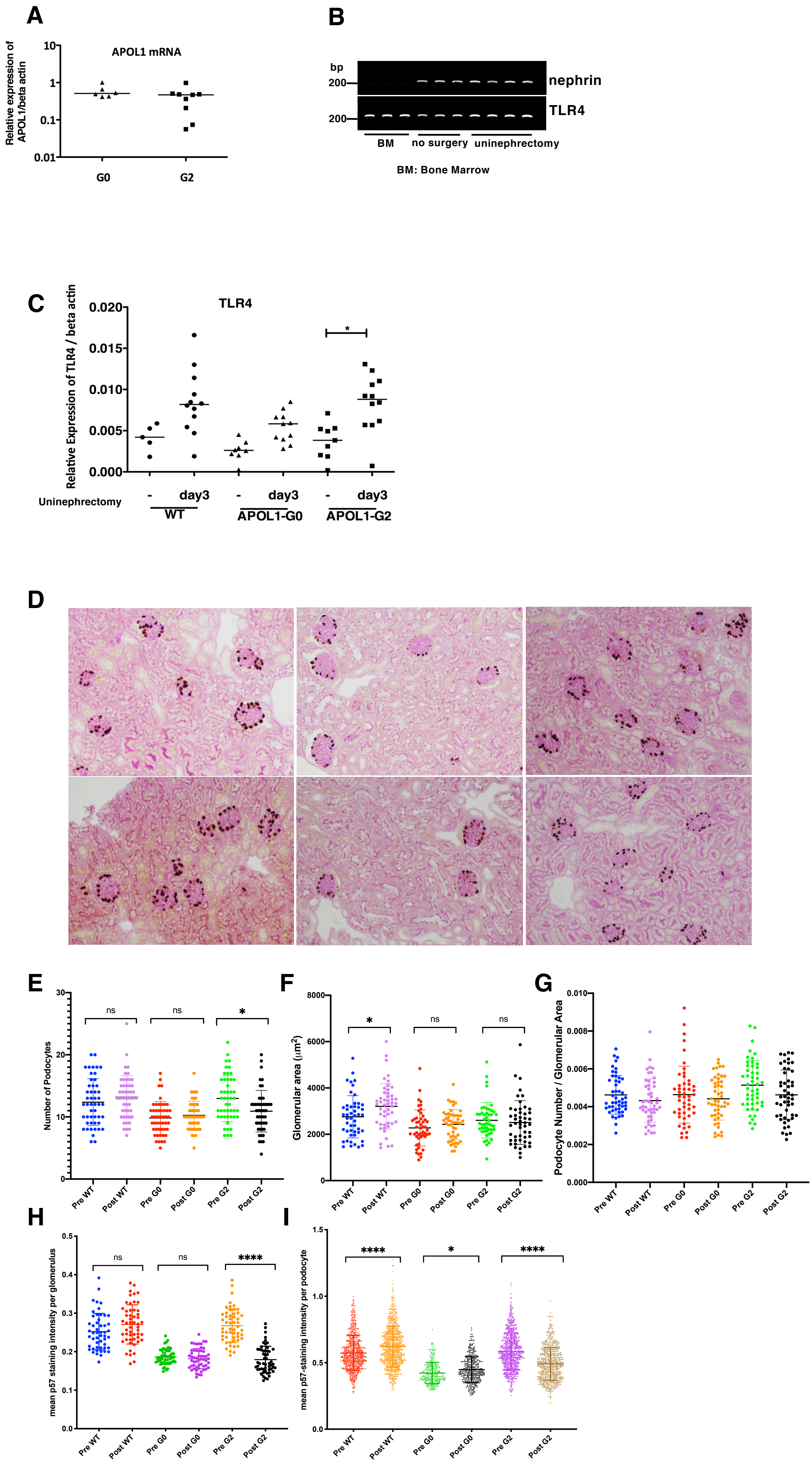

Supplementary Figure 4. TLR4 was upregulated in the remnant kidney after uninephrectomy. (**A**) Expression of APOL1 mRNA from isolate glomeruli was similar in APOL1-B3-G0 and –G2 mice after uninephrectomy. G0: N=6, G2: N=9. APOL1 expression is shown relative to actin. (**B**) Following uninephrectomy, nephrin and TLR4 were expressed in glomeruli isolated from CAG-APOL1-B3 mice. Bone marrow cDNA was used as a positive control for TLR4 and as a negative control for nephrin. (**C**) Glomerular TRL4 mRNA was upregulated following uninephrectomy in all groups and reached statistical significance compared to baseline in APOL1-B3-G2 transgenic mice. **(D**) Immunohistochemistry of formalin-fixed, paraffin-embedded kidney cortex section from wild-type (FVB), APOL1-B3-G0, and APOL1-B3-G2 mice before and after uninephrectomy showing staining for p57 of (from left to right, top row: pre-uninephrectomy; bottom row: post-uninephrectomy): wild-type, APOL1-B3-G0, and APOL1-B3-G2. Quantitative analysis showing, for each group, (**E**) the number of podocytes, (**F**) area of glomeruli, (**G**) ratio of podocyte number over glomerular area, (**H**) p57 staining intensities per glomerular area, and (**I**) p57 staining intensities per podocytes. (ns, not significant; * < 0.03322; **** < 0.0001) APOL1-FLAG

Supplementary Figure 5.

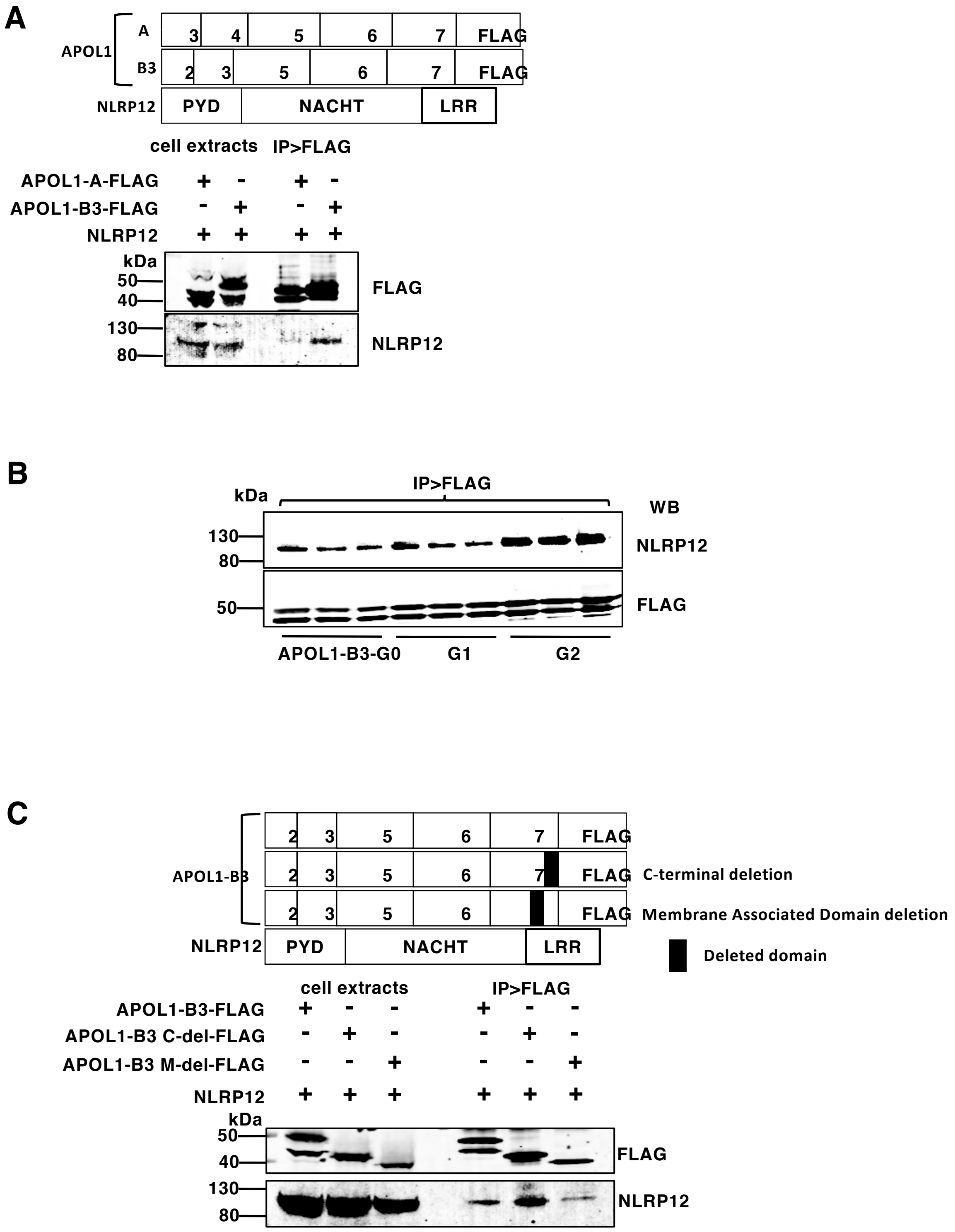

Supplementary Figure 5. Renal risk variant APOL1 associated with NLRP12.
(**A**) The schematic depicts the exon composition of the APOL1-FLAG expression constructs and the domain architecture of NLRP12, respectively. Pull-down experiments of APOL1-NLRP12 cotransfected HeLa cells with anti-FLAG antibodies showed in immunoblots that APOL1-B3 but not APOL1-A interacted with NLRP12. (**B**) NLRP12 and FLAG-tagged APOL1-B3 variants (G0, G1, G2) were transfected into HeLa cells, followed by immunoprecipitation with anti-FLAG antibody and western analysis for NLRP12. (**C**) To further characterize the interactions with APOL1-B3 with NLRP12, we showed similarly that deletion of the C-terminal domain (C-del) and the membrane-addressing domain of APOL1-B3 interacted with NLRP12. APOL1-B3 C-terminal domain was not required in the interaction with NLRP12.

Supplementary Figure 6.

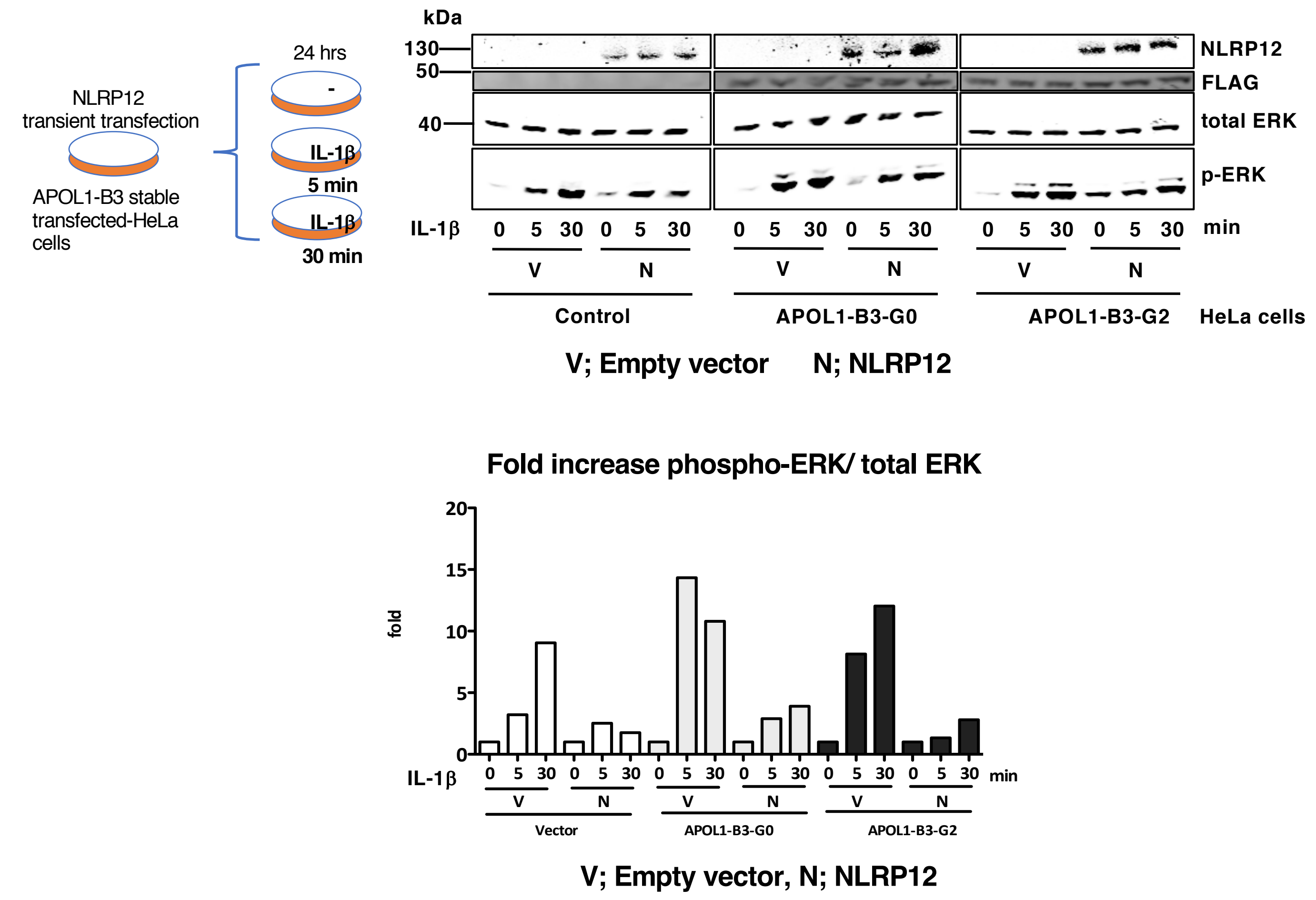

Supplementary Figure 6. Overexpression of NLRP12 antagonized APOL1-B3.
Compared to vector (V) control, overexpression of NLRP12 (N) suppressed phospho-ERK induced by exposure to IL-1β (15 ng/ml) for 5 or 30 min in the presence of APOL1-B3-G0 and APOL1-B3-G2. The expression levels of FLAG-APOL1-B3 protein were similar between groups, confirming equivalent transfection efficiencies. This experiment was repeated twice with reproducible results. In western blot image, protein were loaded different two gels, proteins were transferred to one membrane. The resulting image was trimmed and is shows in one panel.
