# Supplementary material for: APOL1 renal risk variants exacerbate podocyte injury by increasing inflammatory stress
Source: BMC Nephrol. 2020 Aug 27;21:371. doi: 10.1186/s12882-020-01995-3 (PMC7450955; doi:10.1186/s12882-020-01995-3)
Supplement: Supplementary file 2 — Additional file 2. Supplementary methods. Table S1. Antibodies and nuclear staining. Table S2. Primers used for cloning APOL1 splice variants. Table S3. Primers use for RT-PCR, qPCR and genotyping. Table S4. Primers used for sub-cloning of APOL1-FLAG expression vector. Table S5. Primers used for sub-cloning of CAG-APOL1-B3 expression vector. Table S6. Primers used for mutagenesis of APOL1-B3 FLAG vectors. Table S7. Primers used to generate NRLP12 expression vectors. Table S8. Number of mice used in Fig. 5. [file 12882_2020_1995_MOESM2_ESM.pdf]

Supplementary information

- table 1-8
- Supplementary methods

Table 1. Antibodies and nuclear staining.

Target	Peptide	Species	Supplier	Catalog number
APOL1	C terminal residues 125 aa	Rabbit pAb	Sigma	HPA 018885, Lot E96151
APOL1	C terminal residues 125 aa (CL0171)	Mouse mAb	Sigma	AMAB90530
APOL1	NA (EPR2907)	Rabbit mAb	Abcam	Ab108315
APOL1-B isoform	MRFKSHTVELRRPCDS	Rabbit pAb	KDB, NIDDK	APOL1-B
p44/42 MAPK(Erk1/2)	NA	Mouse mAb	Cell Signaling	9107
Phospho-p44/42 MAPK (Erk1/2)	(D13.14.4E) XP (Thr202/Tyr204)	Rabbit mAb	Cell Signaling	4370
NLRP12	654-727 aa	Rabbit pAb	Sigma	HPA042981, Lot R41168,
FLAG M2	DYKDDDDK	Mouse mAb	Sigma	F1804
FLAG	DYKDDDDK	Rabbit pAb	Cell Signaling	2368
Podocalyxin	Ser21-Arg402	Goat pAb	R and D	AF1556
Wilms tumor1	6F-H2	Mouse mAb	Millipore	05-753
Mouse IgG	NA	Donkey, Alexa Fluor 488	Invitrogen	A21202
Rabbit IgG	NA	Donkey, Alexa Fluor 488	Invitrogen	A21206
Nuclei	NA	Hoechst	Invitrogen	33342

Legend. Data for the immunizing peptides are shown when relevant. APOL1-B antibody: APOL1 exon2-exon3 specific peptides (MRFKSHTVELRRPCDS) were used to immunize rabbits and the antibody was affinity-purified. Abbreviations: mAb, monoclonal antibody. pAb, polyclonal antibody. aa, amino acid

Table 2. Primers used for cloning APOL1 splice variants.

TA cloning primers		
V1, V2-1, V2-3, V3 and V4	Forward	ATCTTGCTCAGTCTCTGCCAGG
	Reverse	CCTCCCAATGCTAAACTGCTTTC
V2-2 and V2-3	Forward	TGAGATTCAAAAGCCACACTG
	Reverse	CCTCCCAATGCTAAACTGCTTTC

Legend. Upper primers were used for cloning of APOL1 splice variants V1, V2-1, V2-3, V3 and V4, and lower primers were used for V2-2 and V2-3.

Table 3. Primers use for RT-PCR, qPCR and genotyping.

RT-PCR, qPCR, genotyping		
APOL1 Exon1/5 RT-PCR	Forward	ATCTTGCTCAGTCTCTGCCAGG
	Reverse	CAGTATCTGTCCCACTTGGAACG
APOL1 Exon2/5 RT-PCR	Forward	TGAGATTCAAAAGCCACACTG
	Reverse	CGAGGGGCTTACTTTGAGGA
APOL1 V2-3 variant qPCR	Forward	TGAGATTCAAAAGCCACACTG
	Reverse	GGAACGTTTTGTTGCACCCAG
APOL1 V1 variant qPCR	Forward	GCCAGGGGAAGATTCTTGCGCCTCGCC
	Reverse	ACACCAAGGAAAAGTGCACTCATC
APOL1 genotyping	Forward	CAATGTGGTGCTTGGCTCTCTC
	Reverse	AATGCCTCGTGTTGAGTTGGTAAG
Mouse Nephrin RT-PCR	Forward	ACCTGTATGACGAGGTGGAGAG
	Reverse	TCGTGAAGAGTCTCACACCAG
Mouse TLR4 RT-PCR, qPCR	Forward	CAGCAAAGTCCCTGATGACATTC
	Reverse	CCACAGCCACCAGATTCTCTAAAC
Mouse IL-1β qPCR	Forward	AAGGAGAACCAAGCAACGAC
	Reverse	AACTCTGCAGACTCAAACTCCAC
Mouse β-actin qPCR, RT-PCR	Forward	ACTGCTCTGGCTCCTAGCAC
	Reverse	CAGCTCAGTAACAGTCCGCC
Human GAPDH qPCR	Forward	GGAAGGTGAAGGTCGGAGTC
	Reverse	CAAGCTTCCCGTTCTCAG

Legend. Indicated primers were used for RT-PCR, qPCR and genotyping. 'APOL1 V2-3, V1' is V2-3 or V1 specific primers and 'APOL1' was used for genotyping of APOL1-B3 and BAC APOL1 transgenic mouse.

Table 4. Primers used for sub-cloning of APOL1-FLAG expression vector.

APOL1-FLAG tagged vector		
primer1 V1	Forward	ACTGAATTC ATGGAGGGAGCTG CTTTGCTG
primer2 V2-1, V2-2, V2-3	Forward	ACTGAATTCAATCATGAGATTCAAAAGCCACACTG
Primer3 all variants	Reverse	ACTGGATCCCAGTTCTTGGTCCGCCTGCAGAATC

Legend. APOL1 variants ORF are amplified by (primer1, primer3) (primer2, primer3) respectively. APOL1 variants ORF was inserted into Flag vector multiple cloning sites at EcoR1 and BamH1. The 3X Flag-vector was purchased from Sigma (# E4901).

Table 5. Primers used for sub-cloning of CAG-APOL1-B3 expression vector.

CAG-APOL1-FLAG tagged episomal vector		
APOL1 V2-3 sub cloning	Forward	ACTGGTACCAATCATGAGATTCAAAAGCCACACTG
	Forward	ACTGCGGCCGCCTTGTCATCGTCATCCTTGTAGTCG

Legend. The pEBMulti vector was purchased from WAKO (# 057-08131). APOL1- B3-G0-Flag was amplified with the primers as shown. The DNA was inserted into pEBMulti vector MCS at the *Kpn*1 and *Not*1 cloning sites.

Table 6. Primers used for mutagenesis of APOL1-B3 FLAG vectors.

APOL1-B3-G0-FLAG vector mutagenesis		
G1（p. S342G）	Forward	GGCTTCTTTCTTGTGCTGGATGTAG
	Reverse	TACAGGGGCCACATCCGTGAGCTTG
G1（p. I384M）	Forward	GCTCAACAATAATTATAAGATTCTG
	Reverse	ATGTTTAGCTTCTCCTCCAGCTCC
G2（p. NYK388K）	Forward	AGATTCTGCAGGCGGACCAAGAACT
	Reverse	TATTGTTGAGAATGTTTAGCTTC
residues 357 to 396 deletion	Forward	GACTACAAAGACCATGACG
	Reverse	GTGCTTTGATTCGTACACGAG
residues 236 to 307 deletion	Forward	ATCTCAGCTGAAAGCGGTGAAC
	Reverse	TTGTGTCCACCACTTCTTTCC

Legend. APOL1 renal risk variants vector and domain deletion vectors were generated by indicated primers.

Table 7. Primers used to generate NRLP12 expression vectors.

NLRP12 expression vector		
NLRP12	Forward	ACTGCGGCCGCACCCCCATGCTACGAACCGCAGGCAG
	Forward	ACTGGTACCGCAGCCAATGTCCAAATAAGG

Legend. Human NLRP12 vectors were generated by indicated primers.

Table 8. Number of mice used in Figure 5

Figure 5A (ACR: pre/ post uninephrectomy)

pre/ post Uninephrectomy	WT	G0	G2
F (female)	16	5	2
M (male)	8	7	17
total	24	12	19

Figure 5B (mRNA expression)

Uninephrectomy	WT	G0	G2
F	8	5	3
M	4	6	9
total	12	11	12

no -treat	WT	G0	G2
F	0	7	3
M	5	1	6
total	5	8	9

Figure 5 C (IL-1b in the urine)

Uninephrectomy	WT	G0	G2
F	1	3	2
M	6	5	7
total	7	8	9
base line			
F			1
M			3
total			4

Supplementary methods

TA-cloning of APOL1 variants

Total RNA was obtained from human immortalized podocytes with QiAzol (Qiagen) and cDNA was synthesized by GoScript Reverse Transcription System (Promega). RT-PCR was performed using APOL1 specific primers, as shown in supplemental information, using PrimeSTAR GXL DNA Polymerase (Clontech). Adenine was added to the 3’ end of PCR products, using Ex Taq (Clontech) and dATP (New England Biolabs). Products were inserted into TA vector, pGEM-T Easy Vector Systems (Promega). Competent cells (One Shot TOP10 Chemically Competent *E. coli,* Invitrogen) were transformed by the plasmids and specific clones were selected by blue-white selection. DNA sequences were determined by Eurofins MWG Operon.

Generation of APOL1-B3 stably transfected HeLa cell lines.

CAG-ApoL1-B3-FLAG vectors were transfected into HeLa cells with Lipofectamin 2000 (Invitorogen) and the cells were selected with 500 μg/ml of G418 (Corning) for 14 days. Stable clones were obtained by limited dilution method.

Transient transfection

APOL1 isoform vector (see Supplementary tables) was transfected into immortalized human podocytes with Lipofectamine2000 and incubated at 33˚C. Cells were lysed in 1% NP-40 buffer, and extracts were subjected to western analysis using APOL1-B antibody. Empty vector or NLRP12 vector (1 μg) were transiently transfected into APOL1-B3 stable HeLa cells with Lipofectamin2000.

RT-PCR, qPCR

Total RNA was obtained from mouse kidney and glomeruli with TRizol (#15596026 Invitrogen). The cDNA was synthesized by GoScriptReverse Transcription System (Promega). cDNA from various human tissue were purchased from a commercial source (Human MTC Panel 1 #636742 Lot #1105031A Clontech). cDNA were subjected to qPCR by using primers indicated in supplementary information. qPCR was performed using ABI prism7500HT. Results of qPCR show relative expression [$2^{(\text{Ct house keeping gene-Ct target gene})}$].

IL-1β ELISA

Mouse urine IL-1β was measured by ELISA (R & D Systems) IL-1β values were standardized by urine creatinine (Exocell).

Immunoprecipitation

APOL1-G0-FLAG or empty vector stably-transfected HeLa cells were lysed with Pierce IP lysis buffer after treatment with cross-linker dithiobis (succinimidyl propionate) (DSP) (Sigma Aldrich) at 4 mM final concentration for 30 min on ice. FLAG-tagged APOL1-B3 was immunoprecipitated with FLAG antibody (dilution 1:100) using magnetic bead method (Pierce Classic Magnetic IP/Co-IP kit, Thermo Fisher Scientific). Eluted samples were subjected to mass spectrometry analysis.

Vectors (see Supplementary tables) were generated for expression of APOL1 mRNA variants and NLRP12. Primers used for mutagenesis are listed in Supplementary tables. APOL1 and NLRP12 vectors were dually transfected into HeLa cells with various combinations using Lipofectamin3000 (Invitrogen). Transfected APOL1-FLAG proteins were immunoprecipitated by using anti-FLAG antibody (1:100) and magnetic beads method without cross-linking (Pierce Classic Magnetic IP/Co-IP kit, Thermo Fisher Scientific). Eluted samples were subjected to western analysis.

Western analysis

Protein (20-50 μg) was subjected to SDS-PAGE. Protein was transferred to PVDF or nitrocellulose membranes, and analyzed by using antibodies as indicated in Supplementary information. LI-COR Odyssey imaging system (LI-COR Biosciences) was used for the signal detection. Data were analyzed by MacImageStudioLite-3.1.4.

Histology

Formalin fixed, paraffin-embedded sections, were subjected to citrate buffer antigen retrieval and stained with primary antibodies for overnight at 4˚C in a dilution value 1: 200 (APOL1 antibodies or WT1 antibody) or 1:800 (podocalyxin). Images were obtained by confocal microscopy (Zeiss LSM780) (Figure 2, Supplementary Figure 2 and Figure 4).
